# [^18^F]‐Sodium Fluoride PET/MR Imaging for Bone–Cartilage Interactions in Hip Osteoarthritis: A Feasibility Study

**DOI:** 10.1002/jor.24443

**Published:** 2019-08-30

**Authors:** Radhika Tibrewala, Emma Bahroos, Hatef Mehrabian, Sarah C. Foreman, Thomas M. Link, Valentina Pedoia, Sharmila Majumdar

**Affiliations:** ^1^ Department of Radiology and Biomedical Imaging University of California, San Francisco San Francisco California

**Keywords:** bone remodeling, bone shape, hip osteoarthritis, PET/MRI, *T*_1*ρ*_/*T*_2_ relaxation times

## Abstract

This study characterized the distribution of [^18^F]‐sodium fluoride (NaF) uptake and blood flow in the femur and acetabulum in hip osteoarthritis (OA) patients to find associations between bone remodeling and cartilage composition in the presence of morphological abnormalities using simultaneous positron emission tomography and magnetic resonance imaging (PET/MR), quantitative magnetic resonance imaging (MRI) and femur shape modeling. Ten patients underwent a [^18^F]‐NaF PET/MR dynamic scan of the hip simultaneously with: (i) fast spin‐echo CUBE for morphology grading and (ii) *T*_1*ρ*_/*T*_2_ magnetization‐prepared angle‐modulated partitioned *k*‐space spoiled gradient echo snapshots for cartilage, bone segmentation, bone shape modeling, and *T*_1*ρ*_/*T*_2_ quantification. The standardized uptake values (SUVs) and Patlak kinetic parameter (*K*_pat_) were calculated for each patient as PET outcomes, using an automated post‐processing pipeline. Shape modeling was performed to extract the variations in bone shapes in the patients. Pearson's correlation coefficients were used to study the associations between bone shapes, PET outcomes, and patient reported pain. Direct associations between quantitative MR and PET evidence of bone remodeling were established in the acetabulum and femur. Associations of shaft thickness with SUV in the femur (*p* = 0.07) and *K*_pat_ in the acetabulum (*p* = 0.02), cam deformity with acetabular score (*p* = 0.09), osteophytic growth on the femur head with *K*_pat_ (*p* = 0.01) were observed. Pain had increased correlations with SUV in the acetabulum (*p* = 0.14) and femur (*p* = 0.09) when shaft thickness was accounted for. This study demonstrated the ability of [^18^F]‐NaF PET‐MRI, 3D shape modeling, and quantitative MRI to investigate cartilage‐bone interactions and bone shape features in hip OA, providing potential investigative tools to diagnose OA. © 2019 The Authors. *Journal of Orthopaedic Research* ^®^ published by Wiley Periodicals, Inc. on behalf of Orthopaedic Research Society J Orthop Res 37:2671–2680, 2019

Osteoarthritis (OA) is the most common debilitating joint disease, affecting more than 25% of the adult population. The etiology of OA is multi‐factorial and includes joint injury, aging, obesity, and hereditary factors.^1^ Hip OA is characterized by a number of pathological changes, including progressive proteoglycan loss and collagen disruption in the early stages, destruction of the articular cartilage, thickening of the subchondral bone, osteophyte formation, and bone deformation in the later stages.^2^, ^3^, ^4^, ^5^, ^6^, ^7^ As symptoms of OA do not manifest until the later more painful stages, hence, there is a need for establishing biomarkers to detect OA in its early, reversible stages. Currently, OA progression is evaluated by measuring joint space narrowing by radiograph based Kellgren–Lawrence (KL) scores, Croft scores, and minimal joint space width;^8^, ^9^ however, proteoglycan depletion and water loss occur before joint space narrowing. Similarly, early changes in cross‐talk between early bone remodeling and cartilage changes have been implicated in the development of cartilage lesions, joint degeneration, and the progression of OA.^10^

*T*_1*ρ*_ and *T*_2_ relaxation times provide a measure of proteoglycan content and collagen orientation, respectively, reflecting the cartilage biochemistry and composition.^11^, ^12^ In a previous longitudinal study, patients with hip OA, who demonstrated elongated *T*_1*ρ*_ and *T*_2_ relaxation times, developed morphological cartilage degeneration, indicating that *T*_1*ρ*_ and *T*_2_ relaxation times may be biomarkers for early OA.^13^ The three‐dimensional (3D) proximal femur shape variations were also shown to have associations with morphological and compositional markers of hip joint degeneration as well as associations with demographics.^6^ In addition, studies have shown that [^18^F]‐sodium fluoride (NaF) positron emission tomography and magnetic resonance imaging (PET/MR) is capable of imaging significant bone and cartilage interactions in the knee joint.^14^, ^15^ Associations between pain and tracer uptake were seen in the absence of morphological lesions in the cartilage.^14^ An increase in tracer uptake in the presence of subchondral bone lesions was also observed in the knee, concluding that PET/MR may detect metabolic abnormalities in the subchondral bone before they are seen on MRI.^15^ Although the feasibility of [^18^F]‐NaF PET/MR to image bone cartilage interactions in the knee has been studied, to the best of our knowledge, no previous studies have explored the relationships between bone–cartilage interactions and bone shape in the hip joint using [^18^F]‐NaF PET/MR along with quantitative MR to detect early biomarkers of hip OA.

In attempt to fill this gap, this study aims to: (i) characterize the distribution of tracer uptake and blood flow in the femur and acetabulum in patients with hip OA; (ii) study associations between bone remodeling and cartilage composition in the presence of morphological abnormalities using quantitative MR *T*_1*ρ*_ and *T*_2_ relaxation times, [^18^F]‐NaF standardized uptake value (SUV), and Patlak kinetic parameter (*K*_pat_); and (iii) study bone remodeling by studying the relationship between MR‐based bone shape modeling in the femur and [^18^F]‐NaF‐based bone remodeling and blood flow using SUV and *K*_pat_.

We hypothesized that patients with cartilage lesions would demonstrate higher tracer uptake in the bone surrounding the lesions, indicating bone remodeling accompanying the cartilage degeneration, and that the coxa valga femur shape and cam impingment type abnormalities would show higher tracer uptake on account of bone remodeling.

## METHODS

This is an analytic study (Level of Evidence: III). All subjects provided written informed consent and the study was carried out in accordance with the regulations of the Committee for Human Research at our institution.

### Patient Population

The study was approved by the local institutional review board (IRB) and a written informed consent was obtained from all subjects before the study. All the personnel involved with recruitment, scanning, and analysis of this study were Health Insurance Portability and Accountability Act compliant. Radiographs were obtained prior to the study and Kellgren and Lawrence^3^ grading of the radiographs were performed by a board certified musculoskeletal radiologists with more than 20 years of experience (T.M.L.) to stage the extent of hip OA. The inclusion criteria included: (i) male or female; (ii) no history of hip surgery; (iii) no symptomatic knee OA; and (iv) KL score = 0, 1, 2, and 3. The exclusion criteria included: (i) age < 18; (ii) KL score > 3; (iii) use of an investigational drug for the duration of the study; (iv) history of diseases that may involve the studied joint including systemic inflammatory diseases, crystalline diseases, avascular necrosis, Paget's disease or tumors; and (v) any contraindications to MRI. All subjects also completed the Hip disability and Osteoarthritis Outcome Score (HOOS) survey prior to imaging.^16^

### PET MR Image Acquisition

Image acquisition was performed on SIGNA 3T time of flight (TOF) PET/MR (GE Healthcare, Milwaukee, WI). The timing resolution of this scanner as per the manufacturer was less than 400 ps and a 3D ordered‐subset expectation maximization (OSEM) was utilized for all PET reconstructions.

Subjects prepared with an intravenous catheter were positioned supine feet first, with a large‐size receiver flex coil wrapped around the hip of interest in the PET/MR. [^18^F]‐NaF was used as a tracer, sourced from University of California San Fransico's cyclotron facility, produced using current good manufacturing practices (cGMP) guidelines. Subjects were injected with an average 247.97 ± 19.82 MBq of [^18^F]‐NaF for a dynamic PET scan of 45 min. An effective dose of 0.024 mSv/MBq was used to calculate the radiation exposure to each subject due to the injected [^18^F]‐NaF.^17^ A Dixon fat‐water sequence was acquired for MR‐based attenuation correction of PET photons.^18^ The MR images acquired simultaneously with PET included (i) 3D isotropic CUBE fast spin‐echo and (ii) 3D sagittal combined *T*_1*ρ*_/*T*_2_. MR and PET acquisition parameters are shown in Tables 1 and 2, respectively.

**Table 1:** Magnetic Resonance Imaging Acquisition Parameters

MR Imaging Sequence	Acquisition Parameters	Measurements
Three plane gradient echo		Localizer for choosing coverage
3D FSE (CUBE)	TR/TE = 1200/20 ms, FOV = 15.3 cm, matrix size = 192 × 192, echo train = 32, slice thickness = 0.8 mm	Semi‐quantitative clincal grading of the bone marrow edema and cartilage abnormalities
3D combined T_1*ρ*_/T_2_ MAPSS^19^	TE = 0/10.4/20.8/41.6 ms, TSL = 0/15/30/45 ms, spin lock frequency = 300 Hz, FOV = 14 cm, matrix size = 256 × 128, slice thickness = 4 mm	Cartilage *T*_1*ρ*_/*T*_2_ measurements, shape modeling

3D, three‐dimensional; FSE, fast spin‐echo; FOV, field of view; SHOMRI, scoring hip osteoarthritis with MRI; TE, echo time; TR, repetition time; TSL, time of spin lock.

**Table 2:** PET Acquisition Parameters

Dynamic PET Acquistion Parameters	
Dynamic acquisition time	45 min
Onset after injection	0 min
Number of phases	3
Number of frames/phase	12 frames of 10 s, 6 frames of 30 s, 10 frames of 4 min.
Transverse filter cutoff	6.4 mm
Subset	28
Iterations	4
Matrix	256 × 256
FOV	50 cm

FOV, field of view; PET, positron emission tompgraphy.

### MRI Morphologic Grading

**Figure 1:**
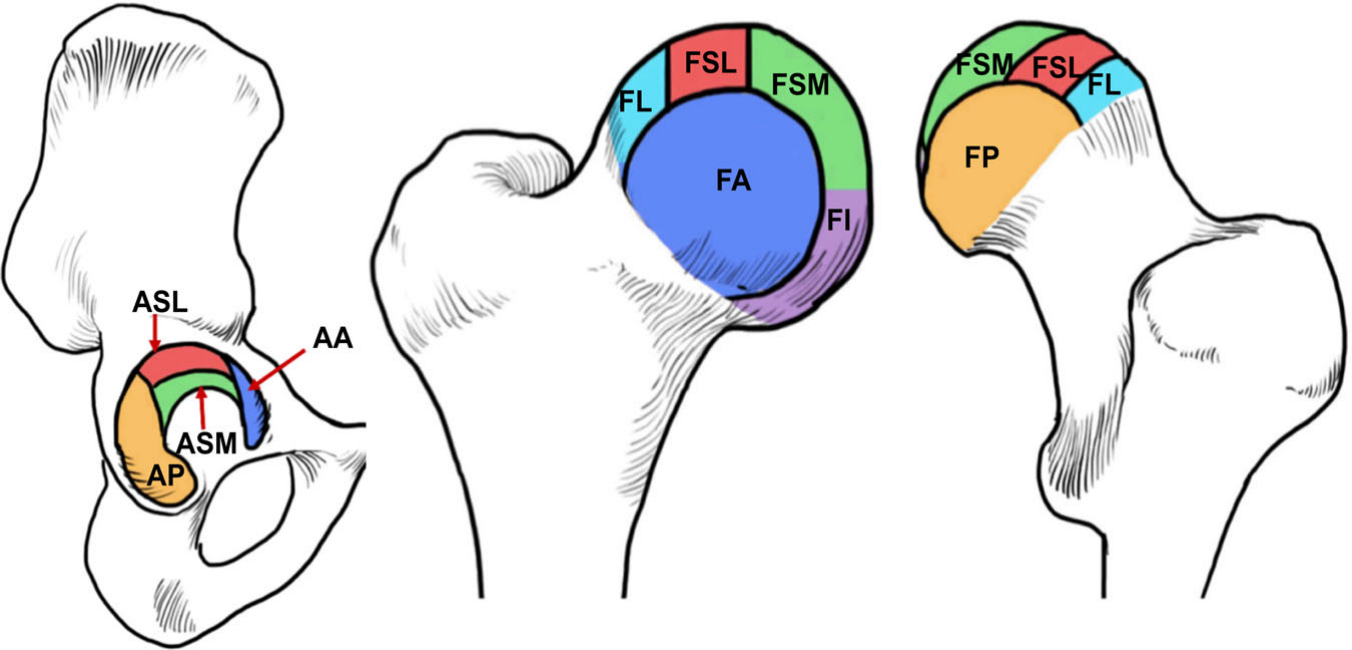
Hip subregions for morphology grading. Sagittal MR images were used to evaluate the AA, FA, AP, and FP subregions. Coronal MR images were used to evaluate ASL, ASM, FL, FSL, FSM, and FI subregions. AA, acetabular anterior; AP, acetabular posterior; ASL, acetabular superolateral; ASM, acetabular superomedial; FA, femoral anterior; FI, femoral inferior; FL, femoral lateral; FP, femoral posterior; FSL, femoral superolateral; FSM, femoral superomedial; MR, magnetic resonance imaging [Color figure can be viewed at wileyonlinelibrary.com]

Semi‐quantitative clinical grading of the abnormalities of the articular cartilage and bone marrow was performed by two radiologists with 3 years of experience, with a Cohen's *κ* value of 0.82 and inter‐rater reliability of 93% between the two readers. All equivocal cases were reviewed by a senior radiologist with more than 20 years of experience (T.M.L.). The 3D isotropic CUBE sequences (CUBE, Table 1) with sagittal, axial, and oblique axial reconstructions were used, which were similar in quality to 2D fat saturated, intermediate weighted sequences, to grade according to a system as described by Lee et al.^20^ Cartilage lesions were graded in 10 subregions using a 3‐point scale: 0 for no loss, 1 for partial thickness loss, and 2 for full thickness loss. The subregions were the acetabular anterior, femoral anterior, and acetabular posterior and femoral posterior cartilage in the sagittal image and the acetabular superolateral, acetabular superomedial, and femoral lateral, femoral superolateral, femoral superomedial and femoral inferior cartilage in the coronal image (Fig. 1). A total cartilage lesion score was obtained for each patient. The highest possible cartilage lesion score was 20. The bone marrow edema (BME)‐like lesion was defined as an ill‐defined subchondral lesion hyperintense on fluid‐sensitive sequences. These lesions were scored in the same 10 subregions using a 4‐point scale: 0 if no lesion was present, 1 if equal to or less than 0.5 cm in size, 2 if greater than 0.5 cm but equal to or less than 1.5 cm, and 3 if greater than 1.5 cm in size. Each of the 10 subregions were scored separately and added for a total bone marrow lesion score. The highest possible bone marrow lesion score was 30.

### Image Processing

Image analysis was performed automatically using an in‐house program developed in MATLAB (Mathworks, Natick, MA). The 3D shape analysis of the femur was performed using the methods as previously described by Pedoia et al, using an atlas‐based method refined by active contours and 3D statistical shape modeling (SSM).^21^, ^22^, ^23^ The SSM has the ability to characterize complex shapes using principal component analysis (PCA). It identifies the shape differences (features) empirically, and does not make any assumptions about the status of OA, therefore, revealing shapes that could have small variations but are nevertheless still related to OA.

For the combined *T*_1*ρ*_/*T*_2_ sequences, non‐rigid registration of all images was performed on a reference space, which was selected as a patient with average body mass index (BMI). *T*_1*ρ*_ and *T*_2_ maps were obtained by fitting the images obtained with different time of spin lock (TSL) and echo time using a Levenberg–Marquardt monoexponential (S(TSL) ∝ e^−TSL/*T*^_1p_), applied to each voxel. The cartilage segmentation, which was performed manually on the reference space was then applied to the *T*_1*ρ*_ and *T*_2_ maps to obtain *T*_1*ρ*_ and *T*_2_ values in the articular cartilage for each patient.^13^

For the PET/MR images, MR images were acquired in the sagittal plane, while PET images were acquired in the axial plane for greater coverage. The axial PET data was first resampled into the *T*_1*ρ*_ (sagittal) coordinates to transform all the PET imges into the same orientation and coverage as the MR images. These transformed images were then registered to the same reference space as used in the MR images, using a nonrigid registration.

Almost all ^18^F‐NaF delivered is retained by bone after a single pass of blood, and is rapidly cleared as a result of bone deposition and kidney excretion.^24^ The arterial input function (AIF) was measured by automatically isolating the signal of femoral artery from the PET data, and was used as an approximation of the plasma input function as the ^18^F‐ions briskly equilibriate with the plasma.^25^ Plotting the PET activity in the dynamic PET images divided by the plasma activity against normalized time results in a straight line after 15 min (equilibration time).^26^ The extraction or exchange efficiency may approach 100% in transfer of fluoride ions from blood to bone, and therefore, the slope of this line (found by fitting Equation (1)) is stated as *K*_pat_ and interpreted as the influx constant, which is a function of the blood flow.^27^ *K*_pat_ has been shown to be proportional to blood flow and bone remodeling in [^18^F]‐NaF studies,^28^ therefore, rendering the *K*_pat_ as a useful metric to model blood flow to the bone (femur and acetabulum). The static PET data was converted to SUV using the injected dose and patient weight.

(1) $$\frac{\text{ROI}(t)}{P(t)}=K_{\text{pat}}\frac{\int_{0}^{t}P(t)\mathrm{dt}}{P(t)}+V_{B}$$

ROI(*t*) is the PET signal measured from a voxel, *P*(*t*) is the plasma input function, and *V*_B_ is representative of the effective volume of the distribution of the tracer.

The entire PET and MR image processing was completely automated and required only the input of all the images of each sequence.

### Individual Case Studies

SUV maps were constructed for each patient and analyzed qualitatively and quantitatively. A radiologist (T.M.L.) identified if the tracer uptake was present in regions, which did or did not have any cartilage or bone marrow lesions. The mean and maximum SUV and *K*_pat_ for each patient along with their mean *T*_1*ρ*_ and *T*_2_ values were calculated, to give a quantitative estimate of interactions between cartilage degeneration and bone remodeling and blood flow.

### Associations of Cartilage and Bone Marrow Lesion Scores With PET Parameters

Overall, Pearson's correlation coefficients were calculated to find associations between total cartilage lesion score, BME lesion score, and quantitative PET parameters (maximum and mean SUV, maximum and mean *K*_pat_) to identify if cartilage and bone marrow lesions can be associated with changes in bone remodeling and blood flow to bone.

### Extraction of Femur Bone Shapes and Their Associations With PET Parameters and Pain

**Figure 2:**
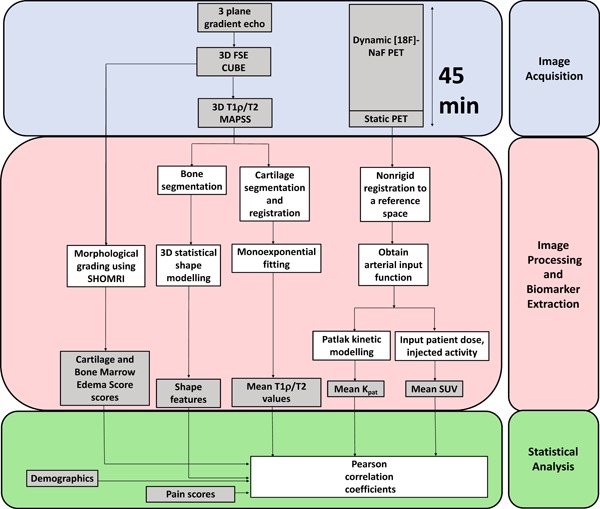
Methods overview. Image acquisition: Three MR sequences were acquired simultaneously with the PET dynamic sequence (45 mins). Image processing and biomarker extraction: The 3D shape modeling, cartilage segmentation, and *T*_1*ρ*_, *T*_2_ values along with the cartilage and BME scores were derived from the MR sequences. The entire PET acquisition was converted to SUV by using the patient weight and injected activity, and a Patlak analysis was performed to determine the rate of net influx of [^18^F]‐NaF. Statistical analysis: All the MR and PET parameters and cartilage and BME scores were inputted into the Pearson correlation analysis. BME, bone marrow edema; MR, magnetic resonance imaging; NaF, sodium fluoride; PET, positron emission tomography; SUV, standardized uptake value [Color figure can be viewed at wileyonlinelibrary.com]

The mean and maximum SUV and *K*_pat_ values were computed for each patient. The 3D shape modeling was performed on 10 patients as described in the methods to extract the principal modes that depict the shape variations in the dataset. Pearson's correlation coefficients were calculated to study the associations between bone shapes and bone remodeling using PET parameters (SUV and *K*_pat_ values). Associations were also studied to see if the PET uptake could correlate with pain. An overview of the entire methods process is contained in Figure 2.

## RESULTS

### Demographics and MRI Findings

Ten patients were recruited for this study, with eight patients (80%) graded with KL scores greater than 0, indicating some sign of hip degenerative changes. The age of the patients ranged from 37 to 77 years (mean = 56.90 ± 12.53 years).

**Figure 3:**
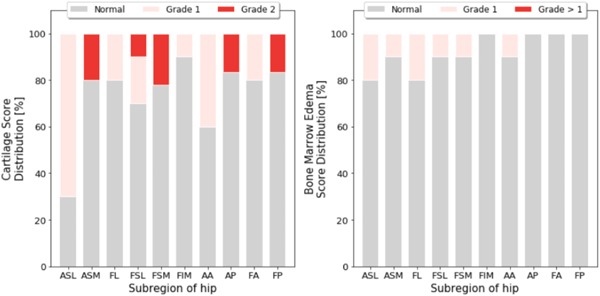
Hip morphology grade distribution. The cartilage score (left) and bone marrow edema score distribution (as %) in the whole patient population as graded in the coronal (ASL, ASM, FL, FSL, FSM, FIM) and sagittal (AA, AP, FA, FP) hip images. AA, acetabular anterior; AP, acetabular posterior; ASL, acetabular superolateral; ASM, acetabular superomedial; FA, femoral anterior; FI, femoral inferior; FL, femoral lateral; FP, femoral posterior; FSL, femoral superolateral; FSM, femoral superomedial; MR, magnetic resonance imaging [Color figure can be viewed at wileyonlinelibrary.com]

Eight patients (80%) exhibited femoral or acetabular cartilage abnormalities and five patients (50%) had BME‐like lesions. The overall demographics of patients are in Table 3 and the distribution of cartilage lesion and bone marrow abnormality scores of the 10 patients in all compartments is in Figure 3.

**Table 3:** Demographics and Clinical Characteristics

Sex^a^				HOOS (0–100, 0 = Worst Outcome)^b^				
Male	Female	Age (years)^b^	BMI (kg/m^2^)^b^	Pain	Symptoms	Quality of Life	Sports/Recreation	Function in Daily Living
8 (80%)	2 (20%)	56.90 ± 12.53	29.00 ± 5.29	92.25 ± 11.87	93.00 ± 11.10	94.11 ± 11.45	93.75 ± 13.50	93.12 ± 12.65

BMI, body mass index; HOOS, Hip disability and Osteoarthritis Outcome Score.

^a^ Represented as percentage (%).

^b^ Represented as mean ± standard deviation.

### Case Studies

While all patients were looked at, three subject analyses are presented in this paper. The rest of the patient images can be found in the supplemental material as a figure. On a single subject basis, an increase in SUV was observed in proximity of structural abnormalities, as well as in places not seen as abnormal on the MRI.

**Figure 4:**
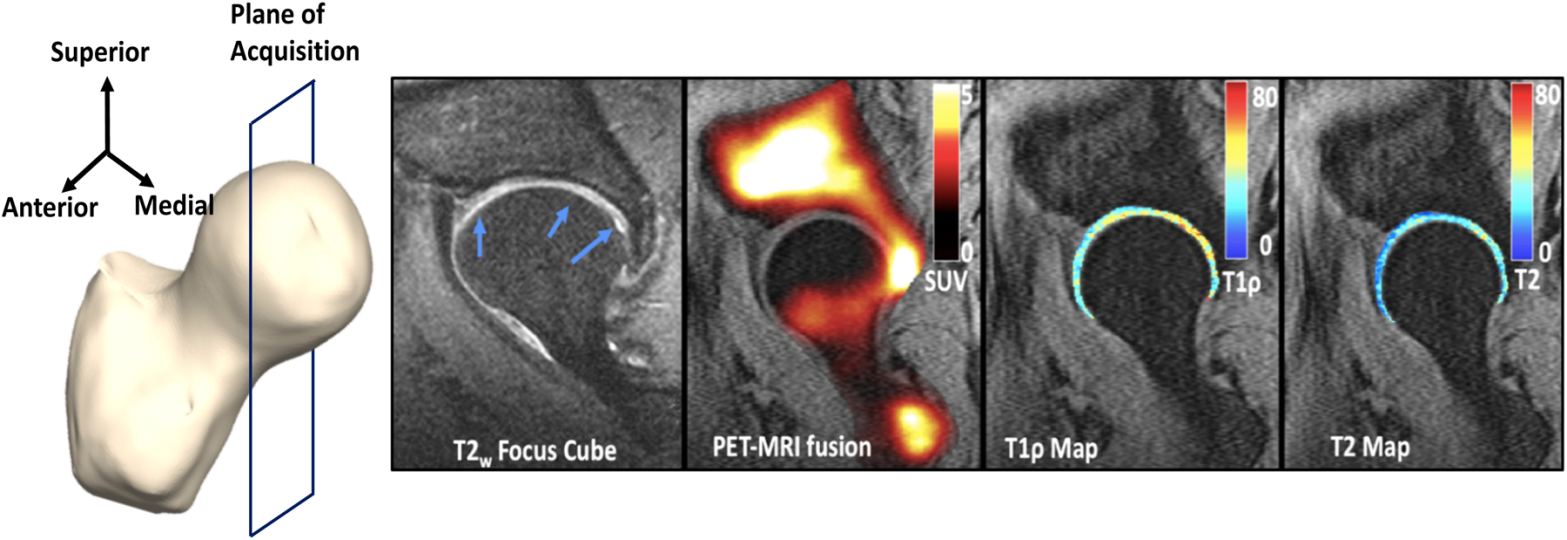
Left to right: Plane of image acquisition, *T*_2_‐weighted focus CUBE image reformatted to a sagittal plane, PET‐MRI fusion image showing all the SUVs (shown in a hot scale: 0 [black] to 5 [white]), *T*_1*ρ*_ map of the articular cartilage using a cartilage mask (shown in a jet scale, ranging from 0 [blue] to 80 [red] ms) overlaid on the first echo of the *T*_1*ρ*_/*T*_2_ sequence, *T*_2_ map of the articular cartilage using a cartilage mask (shown in a jet scale, ranging from 0 [blue] to 80 [red] ms) overlaid on the first echo of the *T*_1*ρ*_/*T*_2_ sequence for a 63‐year‐old male. A very high peak of SUV is seen in the posterior part of the hip near the severe cartilage lesion in the *T*_2_‐weighted image (blue arrow). Adjacent to this region of elevated SUV, elevated *T*_1*ρ*_ and *T*_2_ values are seen. Tracer uptake in the anterior part of the femoral head is seen, which does not seem to correspond to any abnormality. MRI, magnetic resonance imaging; PET, positron emission tomography; SUV, standardized uptake value [Color figure can be viewed at wileyonlinelibrary.com]

Figure 4 shows a *T*_2_‐weighted image showing a cartilage lesion (blue arrows) in the posterior part of the acetabular cartilage in a 63‐year‐old male patient with BMI = 33.98, KL = 2, and pain score = 92.5. This patient had a total cartilage lesion score of 9 out of a maximum score of 20, being the patient with the highest cartilage lesion score. A very high peak of SUV is seen in the posterior part of the hip near the severe cartilage lesion. Adjacent to this region of elevated SUV, elevated *T*_1*ρ*_ and *T*_2_ values are seen, with a mean of 38.13 and 34.62 ms, respectively. These values are higher than the means of *T*_1*ρ*_ and *T*_2_ in all the patients, which are 36.44 ± 3.07 ms and 33.57 ± 3.95 ms, respectively. The elevated morphological values agree with the high cartilage score. There is also tracer uptake in the anterior part of the femoral head, for which a corresponding abnormality is not seen on the MRI. As noticed before, the SUV in the acetabulum is greater than that in the femur, with the mean SUV being 2.42 and 1.90 in the acetabulum and femur, respectively.

**Figure 5:**
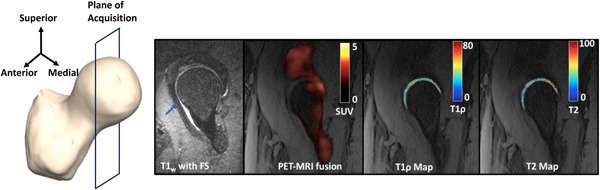
Left to right: Plane of image acquisition, *T*_1_‐weighted sagittal image, PET‐MRI fusion image showing all the SUVs (shown in a hot scale: 0 [black] to 5 [white]), *T*_1*ρ*_ map of the articular cartilage using a cartilage mask (shown in a jet scale, ranging from 0 [blue] to 80 [red] ms) overlaid on the first echo of the *T*_1*ρ*_/*T*_2_ sequence, *T*_2_ map of the articular cartilage using a cartilage mask (shown in a jet scale, ranging from 0 [blue] to 80 [red] ms) overlaid on the first echo of the *T*_1*ρ*_/*T*_2_ sequence for a for a 60‐year‐old male. No uptake is seen corresponding to the region of the edema‐like lesion seen in the *T*_2_‐weighted image (blue arrow). Highly elevated values of *T*_1*ρ*_ and *T*_2_ in the posterior aspect of the joint can be seen in their maps, respectively, with the area of elevation corresponding to SUV elevation location. MRI, magnetic resonance imaging; PET, positron emission tomography; SUV, standardized uptake value [Color figure can be viewed at wileyonlinelibrary.com]

Figure 5 shows a BME‐like lesion (blue arrow) in a 60‐year‐old male OA patient, (BMI = 29.65 kg/m^2^, KL = 2, pain score = 72.5) in the *T*_1_‐weighted sagittal image. No uptake is seen corresponding to the region of the edema‐like lesion seen in the *T*_2_‐weighted image (blue arrow). This patient had a total cartilage lesion score of 5. The mean SUV was 1.22 in the acetabulum and 0.66 in the femur. This difference is visible in the SUV map, where the uptake is much higher in the acetabulum. Highly elevated values of *T*_1*ρ*_ and *T*_2_ can be seen in their maps, respectively, with the area of elevation corresponding to the elevated SUV location. Moderately elevated values of *T*_1*ρ*_ and *T*_2_ are seen in the cartilage corresponding to the edema indicated by the blue arrow. The mean *T*_1*ρ*_ and *T*_2_ values for this patient were 40.33 and 39.25 ms, which were higher than the means of the *T*_1*ρ*_ and *T*_2_ values values of all 10 patients, which were 36.44 ± 3.07 ms and 33.57 ± 3.95 ms, respectively.

**Figure 6:**
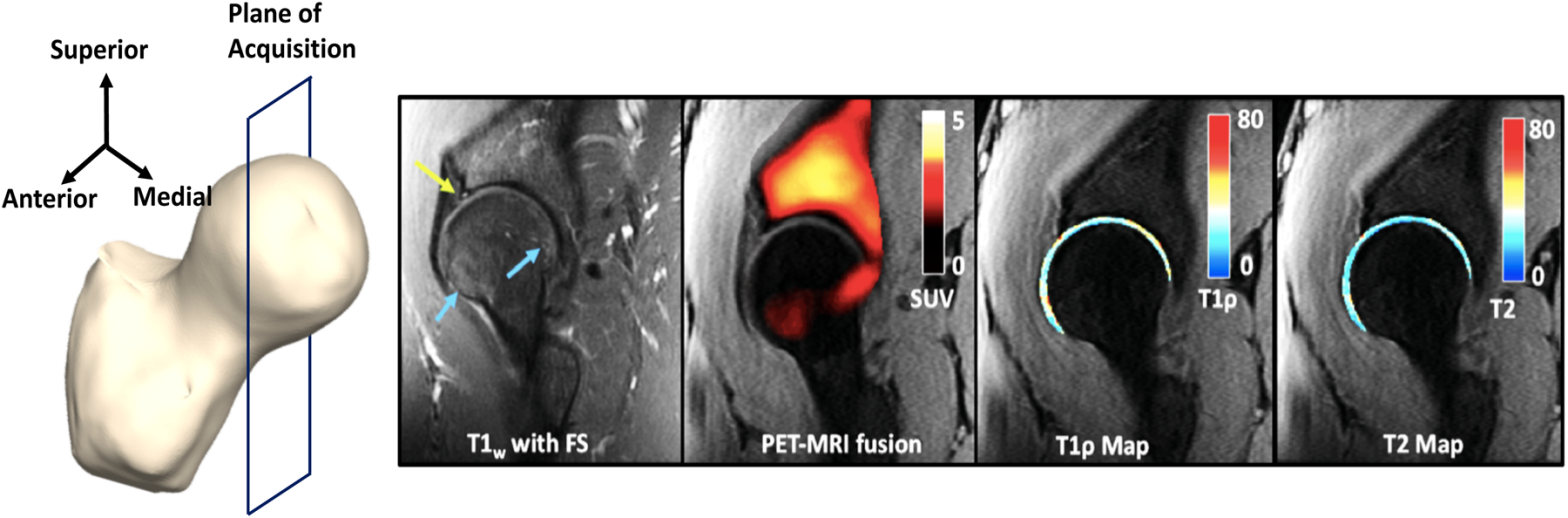
Left to right: Plane of image acquisition, *T*_1_‐weighted sagittal image, PET‐MRI fusion image showing all the SUVs (shown in a hot scale: 0 [black] to 5 [white]), *T*_1*ρ*_ map of the articular cartilage using a cartilage mask (shown in a jet scale, ranging from 0 [blue] to 80 [red] ms) overlaid on the first echo of the *T*_1*ρ*_/*T*_2_ sequence, *T*_2_ map of the articular cartilage using a cartilage mask (shown in a jet scale, ranging from 0 [blue] to 80 [red] ms) overlaid on the first echo of the *T*_1*ρ*_/*T*_2_ sequence for a for a 49‐year‐old male. Bone marrow edema‐like lesions (blue arrows) are seen in the *T*_1_‐weighted image. In addition, this patient had the highest SUV in the acetabulum when compared with other patients, with a value of 10.11. Moderately elevated values of *T*_1*ρ*_ and *T*_2_ are seen in the cartilage corresponding to the edemas indicated by the blue arrows. MRI, magnetic resonance imaging; PET, positron emission tomography; SUV, standardized uptake value [Color figure can be viewed at wileyonlinelibrary.com]

Figure 6 shows BME‐like lesions (blue arrows) in a 49‐year‐old male OA patient (BMI = 21.62 kg/m^2^, KL = 0, pain score = 100) in the *T*_1_‐weighted sagittal image. Even though this patient reported no pain and had KL = 0, his cartilage lesion score was 4. In addition, this patient had the highest SUV in the acetabulum when compared with other patients, with a value of 10.11. As seen in the figure, the uptake in the acetabulum was higher than that in the femur, with a mean of 2.15 in the acetabulum and 1.23 in the femur. Moderately elevated values of *T*_1*ρ*_ and *T*_2_ are seen in the cartilage corresponding to the edemas indicated by the blue arrows. The mean of *T*_1*ρ*_ and *T*_2_ values for this patient were 33.72 and 29.55 ms, respectively.

### Associations of Cartilage and Bone Marrow Lesion Scores With PET Parameters

The SUV for all 10 patients was successfully calculated. The SUV_mean_ in the acetabulum was 2.00 ± 0.76, which was significantly higher than SUV_mean_ in the femur, 1.32 ± 0.47 (*p* = 0.001). The maximum SUV in all the 10 patients was 10.11 in the acetabulum and 3.88 in the femur. Overall, more uptake was observed in the acetabulum as described above.

The *K*_pat_ values for all 10 patients were computed as described in the methods, depicting the effective net influx rate of the tracer. Similar to what was observed for SUV, the mean *K*_pat_ in the acetabulum was higher than in the femur (acetabulum *K*_pat_ = 0.015 ± 0.008 min^−1^ and femoral *K*_pat_ = 0.007 ± 0.005 min^−1^, *p* = 0.002). The maximum *K*_pat_ in all the 10 patients was 0.04 min^−1^ in the acetabulum and 0.03 min^−1^ in the femur.

While, in our single patient analysis we observed cases of discordancy between morphological abnormality and SUV, the BME score showed a high correlation with the SUV_max_ in the acetabulum (*r* = 0.70, *p* = 0.02), but not in the femur (*r* = −0.14, *p* = 0.69). The correlation between the BME lesion score and the SUV_max_ in the acetabulum was further improved when corrected for by age (*r* = 0.79, *p* = 0.006). The total cartilage lesion score did not show any moderate or strong associations with SUV or *K*_pat_ in the femur or acetabulum.

### Extraction of Femur Bone Shapes and Their Associations With PET Parameters and Pain

**Figure 7:**
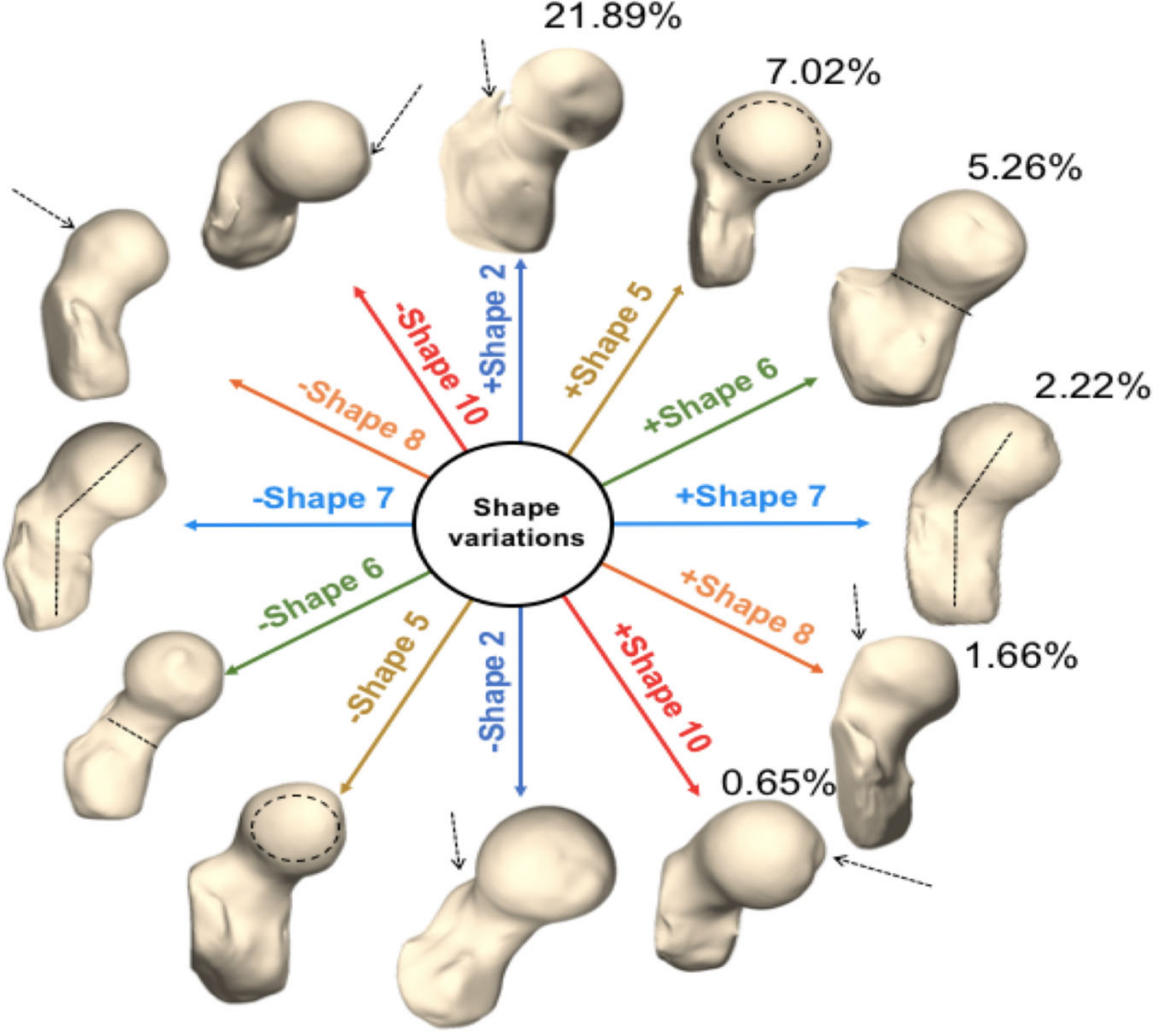
Shape variations derived by 3D SSM in this dataset of 10 MRI exams. Six main shape variations along with the percentage of variation accounted for by each shape are depicted here. Each shape is visualized at mean ± 2 standard deviations. Shape 2 (dark blue): Prominence of the trochanter; Shape 5 (brown): Size of the femur head; Shape 6 (green): Ratio of the radius of the femoral head to the shaft thickness (sub trochanter region); Shape 7 (light blue): Neck shaft angle in the coronal plane; Shape 8 (orange): Cam impingement abnormality in the femur head, Shape 10 (red): Corresponded to a osteophytic growth and deformity on the superomedial part of the femur head (osseus bump). SSM, statistical shape modeling [Color figure can be viewed at wileyonlinelibrary.com]

The principal modes that depict the shape variations in the dataset were extracted. Shape features that showed moderate–strong associations with other imaging data were further explored. Shape 2 described 21.89% of the overall variation within the dataset. Shape 2 described the prominence of the trochanter, with a greater value of shape 2 corresponding to a larger trochanter. Shape 5 (7.02%) described the size of the femur head compared with the neck, with an increasing shape value corresponding to a larger femur head. Shape 6 (5.26%) depicted the ratio of the radius of the femoral head to the shaft thickness (subtrochanter region), with a greater value corresponding to a thicker shaft and smaller femoral head radius. Shape 7 (2.22) described the neck shaft angle in the coronal plane. Higher values of shape 7 correspond to a condition known as coxa valga (increased angle) and lower values of shape 7 correspond to coxa vara (decreased angle). Shape 8 (1.66%) corresponded to a cam impingement abnormality in the femur head, with an increasing value in the shape corresponding to a greater impingement. Shape 10 (0.65%) corresponded to a osteophytic growth and deformity on the superomedial part of the femur head, with a higher value of shape 10 corresponding to a femoral head with more growth and deformity. All the variations in shapes can be visualized in Figure 7.

For both static and dynamic, the coxa valga shape showed stronger correlations in the acetabulum when compared with the femur. It showed a strong positive correlation with SUV in the acetabulum (*r* = 0.70, *p* = 0.02) and weak correlation in the femur (*r* = 0.38, *p* = 0.27). Similarly, with the mean *K*_pat_, the acetabulum showed stronger correlations with coxa valga (*r* = 0.55, *p* = 0.09) when compared with femur (*r* = 0.25, *p* = 0.48). The shaft thickness showed a strong correlation with pain (*r* = 0.81, *p* = 0.004). In addition, the shaft thickness showed a moderate positive correlation with the mean SUV in the femur (*r* = 0.59, *p* = 0.07) and a strong correlation with the maximum *K*_pat_ in the acetabulum (*r* = 0.70, *p* = 0.02). The correlation between pain scores and the maximum uptake was increased when corrected for by shaft thickness (*r* = 0.12, *p* = 0.74 to *r* = −0.50, *p* = 0.14) in the acetabulum and in the femur (*r* = 0.01, *p* = 0.97 to *r* = −0.56, *p* = 0.09). The cam deformity showed weak correlations with PET static and dynamic parameters. The osseus bump on the femoral head showed moderate‐to‐strong positive correlations with the mean femur *K*_pat_ (*r* = 0.69, *p* = 0.01).

## DISCUSSION

This study demonstrated the ability of [^18^F]‐NaF PET‐MRI, 3D SSM, and quantitative compositional MRI to investigate cartilage–bone interactions along with bone shape features in hip OA. Direct associations between morphological MR abnormalities and PET evidence of bone remodeling were established in the acetabulum and femur.

In a case‐by‐case analysis, certain patients were found to have elevated SUV in regions surrounding cartilage lesions, indicating bone remodeling accompanying cartilage degeneration. The BME lesion score showed a high correlation with the SUV in the acetabulum, which increased when corrected for age. For some of the cases, elevated SUV was found in regions that did not depict abnormal (cartilage or other tissue) morphological MRI findings, and these elevations were accompanied by higher *T*_1*ρ*_ and *T*_2_ values. These increased uptakes could be considered early changes occurring in the bone–cartilage matrix that cannot be seen on the morphological MR and may have potential association with pain. Kobayashi et al.^29^ showed that [^18^F]‐fluoride PET can detect bone abnormalities before morphological changes are seen on MRI in hip OA, and that PET uptake may be followed by the appearance of a BME on the MRI. Kogan et al.^15^ showed that [^18^F]‐fluoride uptake was observed in regions that did not correspond to structural abnormalities in the knee, and that it may serve as a marker of early bone remodeling. Interestingly, the highest tracer uptake in the acetabulum was observed in a patient with KL = 0 with no pain. Besides that, no significant differences between tracer uptake, blood flow, or cartilage degeneration were observed between control and other subjects. This may suggest that PET and MR findings are visible in subjects, which have not shown joint space changes in their radiographs yet. In this study, correlations were observed between *K*_pat_ in the femur with the *T*_1*ρ*_ values. These could be indicative of increased blood flow to the femur caused by joint cartilage degeneration. Moreover, we observed an increased *K*_pat_ in regions of the femur where a slight bump was present. Kogan et al.^15^ observed higher SUV in lesions identified as osteophytes in the knee. While we did not see correlations between SUV and osteophytes, the increase in *K*_pat_ in osteophytic regions in the femur could indicate increased blood flow toward these sites. This agreed with Dyke et al.,^30^ who demonstrated that bone blood flow was related to bone formation. We did not see an association between cam deformities and PET static or dynamic parameters, which was unexpected. It could mean that the deformity had formed and was no longer changing or remodeling. However, a longitudinal study is clearly warranted, to determine whether the cartilage degeneration follows the change in femur blood flow or vice versa, that is, if the structural changes in the bone are responsible for the cartilage degeneration or vice versa. A longitudinal study is also required to assess if there are changes in SUV and blood flow during the progression of the osteophyte formation, that is, if the SUV is higher while the osteophyte is still growing and reduces once the osteophyte has fully formed.

While pain scores did not show any strong correlations with MR or PET quantitative parameters, pain scores did show moderate‐to‐strong correlations with different bone shapes, especially shaft thickness, femur head size, and presence of a osseus bump. Previous studies have demonstrated links between certain proximal femur bone shapes in radiographs and OA.^31^ In this study, the coxa valga condition was found to have a strong correlation with the SUV in the acetabulum, which could indicate active bone remodeling in the acetabulum in the presence of cartilage degeneration. Pedoia et al^6^ showed that the coxa valga shape might be a good indicator of early OA progression owing to its correlation with cartilage abnormalities. Pain scores were found to have increased correlations to *T*_1*ρ*_ when the shaft thickness was taken into account, and similarly higher correlations between pain and maximum SUV were observed after incorporating shaft thickness. This indicates that the thickness of the subtrochanter region may be continuously remodeling to adjust bone architecture to meet the needs of the changing joint in osteoarthritic patients, which could manifest as pain and discomfort. A previous study has used multidimensional data analysis in traits of hip OA and shown that pain changes during different stages of the disease.^32^ This further corroborates the need for a longitudinal study, to evaluate pain at different stages of the disease.

This study has promising results, but several limitations need to be considered. First, this was a cross‐sectional study, which did not allow for the temporal order of events to be established. A follow‐up study on the same patients would be needed in order to better establish the progression of OA and the temporal link between bone and cartilage changes in OA. Second, the number of patients recruited was small, and a larger study will have to be performed to see if these correlations and associations are generalizable. A larger study would also be useful in determining the patterns seen in patients that demonstrate tracer uptake in the absence of lesions versus those who show uptake in the presence of lesions. Third, the bone shape analysis was done on low‐resolution images. The shape modeling would be improved if performed on smaller, isotropic voxels, which would allow for smaller deformities and local changes to be detected. Furthermore, having more patients would help identify any shapes that may not be in this dataset, but could still be important for OA prediction. While OA is often characterized and diagnosed by pain, this occurs in later stages of the disease, which are then irreversible. This study was a step to probe hip joint changes on a pathological level in the early stages of the disease, unleashing potential to diagnose hip OA early with possibility for intervention and treatment. Thus, overall, this study provides several insights into the relationship between cartilage biochemistry, bone shape, and bone remodeling in patients with hip OA, and the potential to understand the pathophysiology of disease and directed interventions using these tools are an exciting step forward in the field of OA and musculoskeletal degeneration.^33^, ^34^, ^35^, ^36^

## AUTHOR's CONTRIBUTIONS

R.T., V.P., and S.M.: conception and design. R.T. and V.P.: data processing. E.B. and HM: data collection. S.C. F. and T.M.L.: radiograph, MRI grading. S.M.: obtaining funding. All authors have final approval of the article.

## Supporting information

Supplementary information.
