# Supplementary figures and images for: [18F]‐Sodium Fluoride PET/MR Imaging for Bone–Cartilage Interactions in Hip Osteoarthritis: A Feasibility Study
Source: J Orthop Res. 2019 Aug 30;37(12):2671–80. doi: 10.1002/jor.24443 (PMC6899769; doi:10.1002/jor.24443)

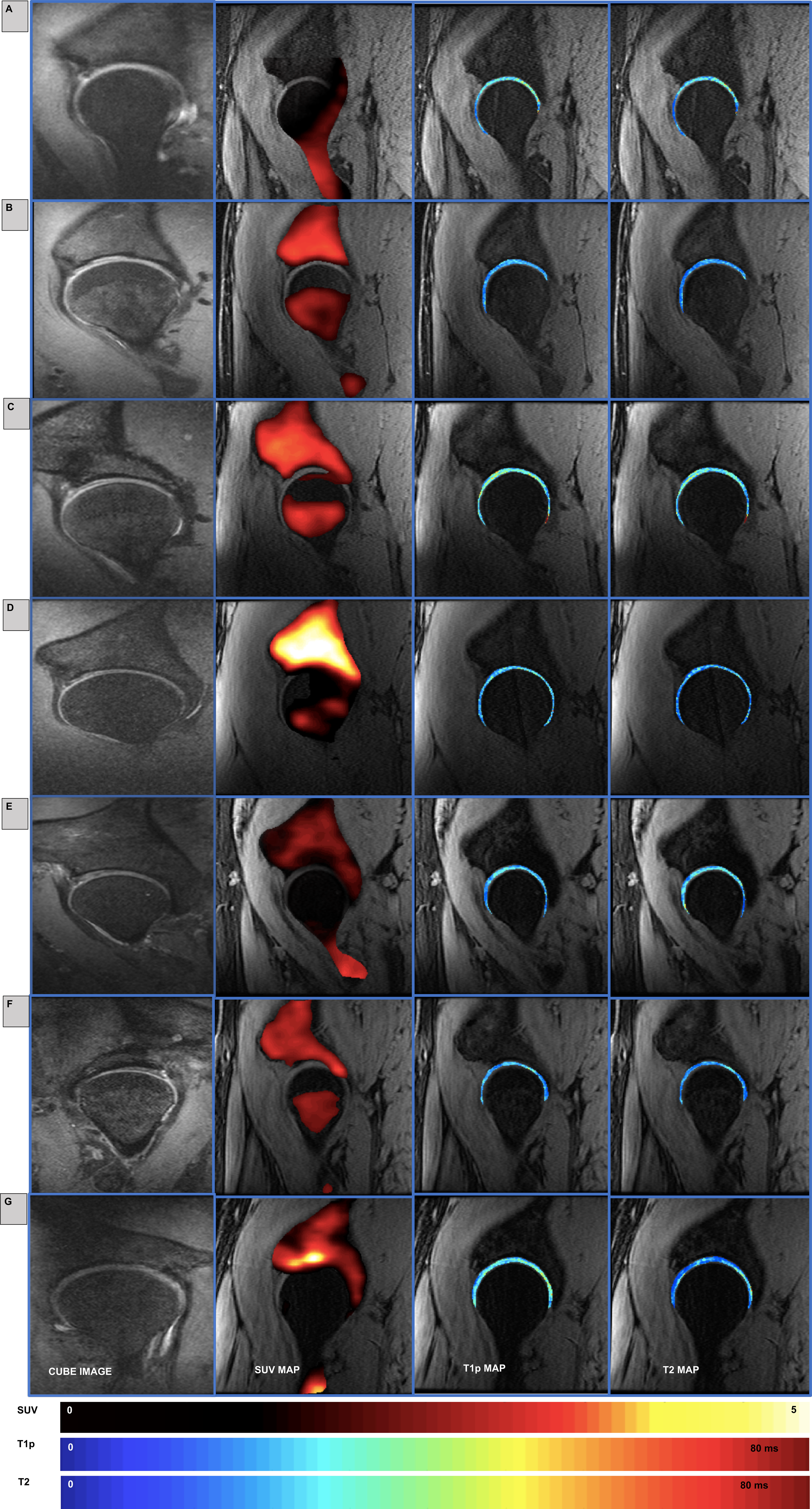

Supplement: Supplementary file 1 — Supplementary information. [file JOR-37-2671-s001.tif]
