# Supplementary material for: [18F]‐Sodium Fluoride PET/MR Imaging for Bone–Cartilage Interactions in Hip Osteoarthritis: A Feasibility Study
Source: J Orthop Res. 2019 Aug 30;37(12):2671–80. doi: 10.1002/jor.24443 (PMC6899769; doi:10.1002/jor.24443)
Supplement: Supplementary file 2 [file JOR-37-2671-s002.docx]

**Supplementary Image:** (In Rows, Top to bottom): Patients (A-G repectively) with age: 67, 38, 55, 37, 77, 61, 62 years, gender: female, male, male, male, male, male, male, BMI: 34.78, 23.24, 36.94, 28.69, 31.42, 23.67, 26.04 kg/m^2^ , KL scores 1, 2, 2, 1, 2, 2, 0 and HOOS reported pain scores: 100, 100, 100, 87.5, 100, 100, and 70. (In Columns, Left to Right): CUBE image reformatted in the sagittal plane, PET-MRI fusion image showing all the SUVs (shown in a hot scale: 0 (black) to 5 (white)), T_1ρ_ map of the articular cartilage using a cartilage mask (shown in a jet scale, ranging from 0 (blue) to 80 (red) ms) overlaid on the first echo of the T_1ρ_/T_2_ sequence, T_2_ map of the articular cartilage using a cartilage mask (shown in a jet scale, ranging from 0 (blue) to 80 (red) ms) overlaid on the first echo of the T_1ρ_/T_2_ sequence.
